# Spatial Thinking in Term and Preterm-Born Preschoolers: Relations to Parent–Child Speech and Gesture

**DOI:** 10.3389/fpsyg.2021.651678

**Published:** 2021-04-23

**Authors:** Sam Clingan-Siverly, Paige M. Nelson, Tilbe Göksun, Ö. Ece Demir-Lira

**Affiliations:** ^1^Department of Psychological and Brain Sciences, University of Iowa, Iowa, IA, United States; ^2^Department of Psychology, Koç University, Istanbul, Turkey; ^3^DeLTA Center, University of Iowa, Iowa, IA, United States; ^4^Iowa Neuroscience Institute, University of Iowa, Iowa, IA, United States

**Keywords:** spatial thinking, children, gesture, parent–child interactions, prematurity, mental transformation

## Abstract

Spatial skills predict important life outcomes, such as mathematical achievement or entrance into Science, Technology, Engineering, and Mathematics (STEM) disciplines. Children significantly vary in their spatial performance even before they enter formal schooling. One correlate of children's spatial performance is the spatial language they produce and hear from others, such as their parents. Because the emphasis has been on spatial language, less is known about the role of hand gestures in children's spatial development. Some children are more likely to fall behind in their spatial skills than others. Children born premature (gestational age <37 weeks) constitute such a risk group. Here, we compared performance of term and preterm-born children on two non-verbal spatial tasks—mental transformation and block design. We also examined relations of children's performance on these tasks to parental spatial language and gesture input and their own production of spatial language and gesture during an independent puzzle play interaction. We found that while term and preterm-born children (*n* = 40) as a group did not differ in the mental transformation or block design performance, children varied widely in their performance within each group. The variability in mental transformation scores was predicted by both a subset of spatial words (*what* aspects of spatial information) and all spatial gestures children produced. Children's spatial language and gesture were in turn related to their parents' spatial language and gesture. Parental spatial language and gesture had an indirect relation on children's mental transformation, but not block design, scores via children's spatial language, and gesture use. Overall, results highlight the unique contributions of speech and gesture in communicating spatial information and predicting children's spatial performance.

## Introduction

Starting from early preschool years, children significantly vary in their performance on spatial tasks (e.g., Levine et al., 1999; Halpern et al., 2007). Although largely ignored in formal schooling, early differences in spatial skills are significant, predicting important life outcomes, such as mathematical achievement or entrance into Science, Technology, Engineering, and Mathematics (STEM) disciplines (e.g., Casey et al., 1997; Benbow et al., 2000; Shea et al., 2001; Wai et al., 2009). Spatial skills are predictors of success in STEM, even after controlling for verbal and mathematical ability (Wai et al., 2009; Uttal et al., 2013). A strong predictor of the individual differences in term children's spatial skills is the spatial language they produce. Children's spatial language is in turn related to the parental input they receive—for example, parents' use of spatial language (Pruden et al., 2011; Levine et al., 2012; Polinsky et al., 2017; Ralph et al., 2020). Some children are at a greater risk of falling behind in their spatial skills (Demir-Lira et al., 2019). Preterm-born children (gestational age <37 weeks) constitute such a risk group. Yet, little is known about the nature of the spatial language children born preterm produce and receive, and the role of parental input in preterm children's spatial development. While prior literature mostly focused on spatial language, hand gestures are tightly linked with spatial thinking as well. The current paper aims to address these gaps in the literature. We compare non-verbal spatial skills of preterm- and term-born children, examine differences in parent and child spatial speech and gesture between the two groups during a puzzle play activity, and finally identify the role of parent and child verbal and gestural input produced during puzzle play in predicting individual differences in children's spatial skills in both groups.

### Spatial Skills in Children Born Preterm

During preschool years, children go through significant developments in their spatial skills (Newcombe et al., 2013; Frick et al., 2014). Among different spatial tasks, some include non-verbal spatial skills such as being able to rotate objects in mental space and replicating spatial patterns. Mental rotation skill has been particularly emphasized in terms of its relation to STEM (Laski et al., 2013). Mental rotation involves mentally imagining, manipulating, and transforming objects (Shepard and Metzler, 1971). Although looking time studies suggest that infants seem to mentally rotate objects (e.g., Moore and Johnson, 2008; Frick and Möhring, 2013; Christodoulou et al., 2016), mental rotation has an extended developmental trajectory, and shows important individual differences (e.g., Estes, 1998; Levine et al., 1999; Okamoto-Barth and Call, 2008; Newcombe, 2020).

Children born preterm, who constitute more than 1 in 10 babies born worldwide (gestational time <37 weeks; Blencowe et al., 2012; Chawanpaiboon et al., 2019), are at a significant risk for falling behind in their visuospatial development (Breslau et al., 1996; Taylor et al., 2000; McGrath and Sullivan, 2002; Anderson et al., 2003; Davis et al., 2005; Marlow et al., 2007). For example, 4-year-old children born extremely preterm (<28 weeks) or very preterm (29–32 weeks) reveal difficulties on visuospatial constructive skills compared to term preschoolers, even after accounting for differences in visual processing, language skills, and demographic factors (Dall'Oglio et al., 2010). Similarly, a number of studies have shown that children born very or early preterm fall behind in their spatial development starting from 4 years of age (Esbjørn et al., 2006; Dall'Oglio et al., 2010). Despite their importance among various spatial skills, very little is known about mental rotation abilities in children born preterm (PTB). To our knowledge, only two studies by Taylor and Jakobson (2009, 2013) used a mental rotation task and reported lower performance in 5–9-year-old very PTB children compared to term (TB) peers. In addition to paucity of research on mental rotation abilities, the emphasis in the literature has been on visuospatial difficulties of extremely and very PTB children. Much less is known about difficulties in PTB children across the full spectrum of gestational age. From a theoretical standpoint, earlier PTBs frequently suffer from neurological impairments or other complications. A focus on the full spectrum will reveal a better characterization of the role prematurity *per se* on spatial development. Thus, the first goal of the current paper is to compare mental rotation skills of TB and PTB children using the full spectrum of gestational age.

### Role of Parental Factors on the Development of Children Born Term and Preterm

Some PTB children fare better than others, which highlights the importance of examining the predictors of the individual variability in PTB children's outcomes over and above group differences. The prior focus has been on the biological risk factors. However, despite the rich knowledge base on the role of biological risk factors, PTB children's outcomes fail to improve. Given this, more recent work on preterm children has emphasized the role of environmental factors in explaining the variability in children's cognitive and academic outcomes. The majority of the literature on the role of environmental characteristics focused on broad characteristics of the environment, such as parental socioeconomic status (SES). For PTB children, parental SES strongly predicts academic outcomes, even more than biological factors such as obstetrical risk (Nepomnyaschy et al., 2012) and moderates the relation between prematurity and academic outcomes (Nomura et al., 2008). However, parental SES is a composite factor consisting of myriad subcomponents (e.g., parental education, income, neighborhood characteristics)—any one of these components could more strongly predict child outcomes than others. Recent work with TB children highlights the role of specific, day-to-day parental cognitive stimulation in predicting children's cognitive development. Among different aspects of cognitive stimulation, parental language input is a strong predictor of child outcomes, over and above parental SES (e.g., Rowe, 2012; Demir et al., 2015).

Less is known about specific aspects of parent–child interactions in PTB children and how these interactions would predict positive outcomes as in TB children. Some studies focused on broad characterizations of parenting, such as parental sensitivity or directiveness (e.g., Foster-Cohen et al., 2010; Lowe et al., 2012; Neel et al., 2018). This body of work shows that PTB children benefit from sensitive, responsive parenting in terms of their socio-emotional and cognitive development. Recent research on term children has emphasized the role of the caregiving environment in the differential susceptibility of children (Belsky et al., 2007). Per the differential susceptibility hypothesis, PTB may be more susceptible to variability in the environment compared to TB—in other words, consequences of negative environmental exposures but also to the benefits of positive ones (Shah et al., 2013; Gueron-Sela et al., 2015). For example, Gueron-Sela et al. (2015) reported that, on a measure of social competence, PTB exposed to high maternal stress performed worse than PTB exposed to low maternal stress. The PTB exposed to low maternal stress even outperformed TB. Overall, although it is clear that parent–child interactions play an important role in PTB children's development, whether the role of the input is the same, lower, or higher remains an open question. With respect to visuospatial development specifically, a longitudinal study by Assel et al. examined the role of parenting style in children's visuospatial skill development (Assel et al., 2003). Parental directiveness at 2 years of age predicted lower visuospatial scores at age 3, which in turn had indirect effects on children's later mathematical development. However, almost nothing is known about the specific interactions that might most closely predict children's spatial skill, such as spatial language, which we discuss next.

### Relations of Parent and Child Spatial Language and Gesture to Child Spatial Development

As argued by Gentner (2016), language presents a “toolkit” to enhance cognition. Specific types of language may also augment certain cognitive processes. Spatial language includes words describing spatial features and properties of the objects, such as *big, tall, edge, up*, and *next to*. Spatial language is argued to influence children's spatial development via carving continuous space into categories and highlighting relevant spatial categories and relations (Roseberry et al., 2012). Spatial language emphasizes spatial information (e.g., Dessalegn and Landau, 2008; Shusterman et al., 2011; Gentner, 2016; Miller et al., 2016), facilitates abstraction of relational commonalities (Casasola, 2005; Loewenstein and Gentner, 2005), or assists children to focus on task-relevant information (Miller and Simmering, 2018). A rich body of literature highlights and presents tight links between children's spatial skills and their spatial language use (e.g., Hermer-Vazquez et al., 2001; Balcomb et al., 2011; Miller et al., 2016, 2020; Levine et al., 2018; Simms and Gentner, 2019; Turan et al., 2021). For example, 4 years-old children's knowledge of the spatial relation “middle” and “between” predicted their search of a hidden object at the midpoint of two landmarks. Children's adaptive use of task-relevant language (both spatial and non-spatial) was also related to a spatial task composite score that included spatial analogies, mental transformation, feature binding, and a picture rotation task (Miller et al., 2016). In a recent study, Turan et al. (2021) found that preschoolers' knowledge of postpositions (a specific type of spatial language) was associated with their mental rotation skills.

Over and above children's own use of spatial language, parent language input about spatial concepts predicts term children's spatial outcomes, controlling for parental SES or general language input quantity (e.g., Pruden et al., 2011). Pruden et al. (2011) reported that the amount of spatial language parents use with their children throughout preschool years relates to children's own spatial language use, which in turn predicts children's performance on spatial tasks at school entrance (Pruden et al., 2011). In other words, researchers reported a mediation model where the role of the parent input on child spatial skill was mediated by children's own spatial language use. Interestingly, the role of spatial language input might also be task specific. In the study by Pruden et al., spatial input of the parents predicted children's mental transformation performance only, but not their performance on another non-verbal spatial task—block design. The results were interpreted to indicate that the role of spatial language input may be more important for tasks where verbalizing a diverse array of spatial features and relations might be needed—as in the case of mental transformation tasks. Block design task requires children to copy patterns consisting of only a small array of spatial elements. Providing causal evidence for the role of input, Polinsky et al. (2017) manipulated parental spatial input during a children's museum exhibit. Higher levels of parental spatial input prompted children to use richer spatial language, which in turn predict children's improvements on a puzzle task.

Parents also use hand gestures in addition to speech when interacting with their children. Parental gestural input predicts later child language skill, with contribution of parent gesture exceeding speech in certain cases (Rowe and Goldin-Meadow, 2009). Spatial topics might be especially conducive to gesture. Gesture is frequently used by adults when talking about space and even is more likely to appear when individuals use spatial words or talk about spatial topics (Krauss, 1998; Emmorey et al., 2000; Sauter et al., 2012). Gesturing during a mental rotation task has been found to improve children's spatial reasoning (Ehrlich et al., 2006). Minimal work focused on parental gesture about specific topics in general, and less is known about parental spatial gestures in TB or in PTB children. Parents' gestures might be particularly enriching for children's language and thinking, because gestures capture continuous features of space better than speech. For example, when talking about a corner piece in a puzzle, parents might reveal the meaning in different ways via gesture—by pointing to the corner of the piece, by tracing the corner with an index finger, and by pointing to both the corner piece and the corner of the puzzle at the same time. Only two studies explored parental spatial gestures. Parental spatial talk accompanied by gestures when children are 12–42 months of age predicted children's own concurrent use of spatial language over and above parental spatial talk without gestures and parental non-spatial talk (Cartmill et al., 2010). However, this study only focused on a subset of spatial relations and, most importantly, did not relate variability in spatial language and gesture use to an independent measure of children's spatial skills. Parents vary in the spatial input they provide to children. Some of the variability is due to children's characteristics, such as age, child language skills, and gender. In a recent study, Kisa et al. reported that parental spatial language might vary as a function of age during toddlerhood and children's own spatial language comprehension assessed at an earlier age (Kisa et al., 2019). To sum, although gesture is tightly linked to spatial thinking, little is known about the role of parental spatial gestures in TB or PTB children's spatial development.

### Current Study

Building on this work, this study has three aims. First, we compare spatial skills (i.e., mental rotation and block design skills) of TB and PTB children, using the full spectrum of gestational age. The second goal is to compare spatial language and gesture of TB and PTB children use as well as the spatial language and gesture input their parents provide during a puzzle play activity. The third goal is to examine the role of child and parental language and gestural input that might predict individual differences in spatial skills of both groups. To address these questions, we focus on spatial language and gestures produced during a puzzle play activity given prior work revealing puzzle play as a rich context for developing mental rotation skill (Levine et al., 2012). One reason might be that completing a puzzle involves both physical and imaginary movements of puzzle pieces. As they transform the pieces, parents and children must determine how to fit the different puzzle pieces together; they can readily observe if the pieces fit or not and thus receive immediate feedback on whether their transformations are accurate. Overall, puzzle activities might serve as a particularly rich context for parents to produce spatial language for their children. Further, puzzles are commonly available in children's home. While some report gender differences in the quantity and quality of play with certain spatial toys, such as blocks, puzzle play does not differ for boys vs. girls (Serbin et al., 1990; Kersh et al., 2008).

Given the paucity of research on both spatial skill across the full spectrum of gestational age and also on specific aspects of input, we do not have strong predictions regarding differences between TB and PTB children and their parents. Per differential susceptibility hypothesis and given prior work suggesting that PTB children might be more open to environmental effects (Gueron-Sela et al., 2015; DeMaster et al., 2019), we expect that parental input will more strongly predict spatial skills in PTB children than TB. Based on work on typically developing children's language (Pruden et al., 2011), we expect a mediation model where parental language and gesture input will predict children's spatial skill via children's own language and gesture use. We use two types of spatial tasks to see if spatial language and gesture are generally predictive of children's spatial performance or specific relations exist—a mental transformation task and a block design task. Given prior findings (Pruden et al., 2011), we expect significant relations to the mental transformation task, but not to the block design task.

## Methods

### Participants

The sample consisted of 40 parent–child dyads from a small Midwestern city in the US. This study was part of a larger study of cognitive development in PTB and TB children. Parent–child dyads were recruited from the University of Iowa Hospital Electronic Health Records (EHR). Parent–child dyads were eligible for this study if they met the following criteria: the child is between the ages of 3 and 5 years old, the child was born at University of Iowa Hospitals and Clinics (UIHC) or received neonatal care at UIHC, the child is a native speaker of English, the child has normal or corrected-to-normal vision and hearing, the child has no physical limitations that would prevent them from completing paper/pencil tasks, and the child has no history of a genetic syndrome, birth defect, or intellectual and developmental disability. Data collection began in June 2019 and ended in March 2020, due to COVID-19-related restrictions. Twenty children born term (gestational age more than 37 weeks) and 20 children born preterm (gestational age 36 weeks 6 days and below) participated in the study (see Table 1 for demographic characteristics). Four of the preterm-born (PTB) children were extremely preterm (born at or before 25 weeks), three were very preterm (born at <32 weeks), five were moderately preterm (between 32 and 34 weeks), and the rest were late preterm (born between 34 and 36 weeks). Children who had data on the measures described below, specifically (1) observations of parent–child puzzle play and (2) child measure of spatial skill, were included in the subsample analyzed here. Parents were overall of higher SES background—average education in years was approximately 16 years, corresponding to a college degree, and average family income was $106,865. Thirteen of the parents reported medical complications during pregnancy, such as preeclampsia, infection, or gestational diabetes. PTB children with and without medical complications did not differ on their spatial scores (CMTT, *U* = *26, p* = *0.15*, WPPSI Block Design, *U* = *33.5, p* = *0.45*). Thus, in the subsequent analysis these groups were combined. All parents provided written informed consent for their family's participation in the study, and all participating children provided verbal assent. All procedures were approved by the University of Iowa's Institutional Review Board.

**Table 1:** Descriptive statistics for demographic characteristics and spatial performance by prematurity group (PTB, TB).

	**PTB**		**TB**			
***n* = 40**	**Mean (%)**	***SD***	**Mean (%)**	***SD***	**t**	***p***
Chronological age (years)	4.47	0.31	4.56	0.28	0.92	0.36
Female ratio	0.40	–	0.55	–	1.67	0.43
Gestation week	31.10	4.51	39.60	1.35	8.19	<0.001^**^
Birth weight (pounds)	3.41	1.94	7.74	1.01	8.87	<0.001^**^
Maternal education (years)	15.40	1.60	16.20	1.82	1.47	0.15
Family income	105,300	57,695	108,429	90,874	0.13	0.89
CMTT	4.33	1.83	5.00	2.16	0.95	0.35
WPPSI block design	10.05	3.39	10.50	2.91	0.44	0.66

*Descriptive statistics include mean and standard deviation (SD). Inferential statistics include t- and p-values.*

** *p < 0.001*.

### Materials

#### Parent Questionnaire

Parents were asked to fill questionnaires on family demographics, parent/child medical history, and other measures, such as parent–child typical daily activities.

#### 24-Piece Puzzle

**Figure 1:**
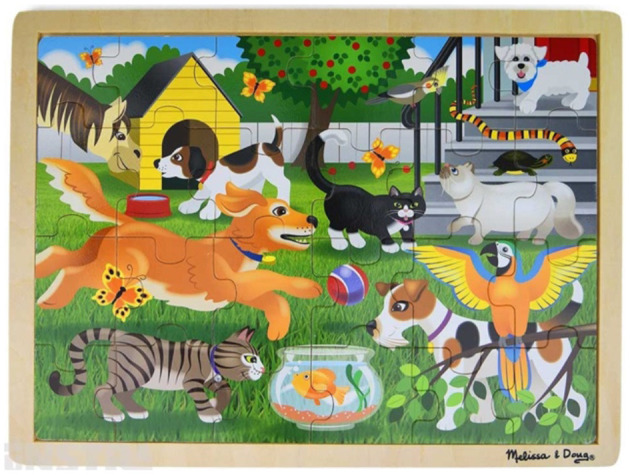
24-piece jigsaw puzzle presented to parent–child dyads in the lab.

Parents were presented a wooden 24-piece jigsaw puzzle to play with (see Figure 1).

#### CMTT Children's Mental Transformation Task

**Figure 2:**
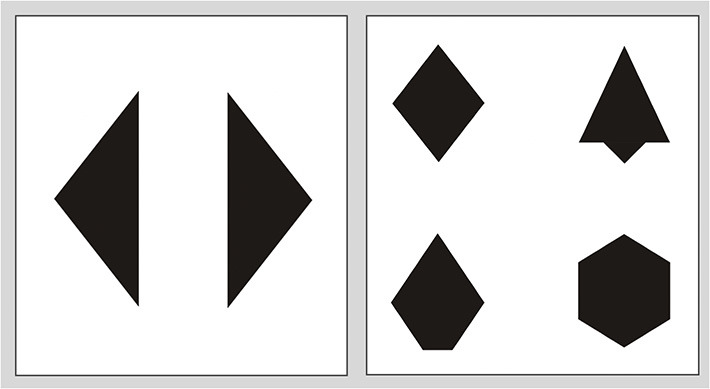
Sample stimuli from the Children's Mental Transformation Task (Levine et al., 1999). Children are asked to pick the shape that the two pieces would make when put together.

Children were administered an abbreviated version of the Children's Mental Transformation Task (CMTT, Levine et al., 1999). This is a non-verbal spatial task that contains 12 trials, assessing children's ability to mentally transform different halves of 2D shapes to make a whole. In this task, children are presented with four shapes and two target pieces (Figure 2). They are asked to select the shape that the two target pieces would make if they were put together. Items varied in the nature of the transformation required−6 trials had items that were rotated 45 degrees from each other and required a rotation of the pieces (rotation items), six trials were translated from each other through a horizontal or diagonal displacement and required translation of the pieces (translation trials). Children were given one practice trial with feedback. Every correct trial received 1 point, and children completed 12 items. Thus, the possible score range was 0 to 12 points. Five of the PTB children and 1 of the TB children were not administered the task due to child fatiguing or experimenter error. Thus, analysis focusing on CMTT was conducted on 34 children only.

#### WPPSI Block Design

Children were also administered Wechsler Preschool and Primary Scale of Intelligence (WPPSI-IV) (Wechsler, 2012). One of the subtests was the Block Design subtest. This subtest is considered to tap into visuospatial constructive processing. In this task, children are presented with blocks with surfaces of solid red, solid white, and surfaces that are half red and half white. They are asked to replicate a pattern the experimenter presents to them, first as a physical model and then with a two-dimensional picture. Children completed 20 questions, and the maximum raw score was 40. One of the PTB children was not administered the task, and analysis on Block Design was conducted on 39 children only.

### Procedure

Parents and children were invited to the lab. Parents were first asked to fill out demographics and background questionnaires. Children were administered a set of experimental and standardized tests by the experimenter in a quiet room, two of which were the CMTT and WPPSI Block Design. Subsequently, parents and children were given three set of bags. Each bag contained a different toy (a puzzle, a book, and a pie sorter) to play with in a quiet room. The activity was modeled after the three bags task where parents were asked to open the bags in order (Nord et al., 2006). Dyads were given a total of 15 min to play with the three sets of toys and were also given a sand-timer to allocate approximately 5 min for each activity. For the purposes of the current paper, only the puzzle activity was analyzed. The order of the toys was counterbalanced across dyads. The interaction was videotaped and transcribed subsequently.

### Parent and Child Speech and Gesture Coding

#### Speech

Parent and child speech during the puzzle interaction was transcribed using the videos. The puzzle interaction was considered to begin when the dyad first interacted with the puzzle pieces. It ended when the puzzle was completed, when the dyad went to the next activity or ran out of time in cases where the puzzle was the last activity. The number of *word tokens* produced during the puzzle activity served as our overall speech measure.

Spatial words parents and children produced were also coded using an adapted version of the System for Analyzing Children's Language about Space (Cannon et al., 2007)—a manual for identifying and categorizing spatial words and phrases in English. Coding was modified based on prior work (Pruden et al., 2011; Kisa et al., 2019) examining the spatial language use in parents and children. We used six categories: (1) *dimensional adjectives* that describe the size of a person, place or thing (e.g., big, tall, little), (2) *spatial feature terms* that describe properties of non-dimensional aspects of objects (e.g., bumpy, corner, flat), (3) *positional and directional terms* that describe the relative position of a person or thing in space (e.g., around, top, between), (4) *shape terms* that are used to label any 2D or 3D enclosed spaces (e.g., circle, sphere), (5) *orientation and transformation terms* that describe the relative orientation or transformation of objects in space (e.g., turn, rotate), and (6) *continuous amount terms* that describe the amount of continuous quantities (e.g., whole, half, part). If the targeted words were used in a non-spatial manner, e.g., *that's a big problem*, they were not considered as a part of the spatial language measure. Similarly, if spatial words were used in a non-task-relevant manner, e.g., *yes, the camera is up there*, these would not have been included in the analysis even when they occurred during the puzzle play. Our measure of spatial language was *spatial tokens* assessed by the number of spatial words used.

#### Gesture

Parents and children's spontaneous gestures produced during the puzzle activity were also identified. The number of gestures produced by the speaker was our overall gesture measure (*gesture tokens*). Gestures consisted of three categories, (1) *deictic gestures* used to indicate a referent such as an object or person in the immediate environment via index finger or palm pointing or via holdups, (2) *iconic gestures* that described an aspect of their referent such as size, shape, or movement (Cartmill et al., 2012), and (3) *tracing gestures* that combined features of deictic and iconic gestures, where the gesturer described an aspect of the reference as they indicated the referent in space. Other main categories of gestures, including beat, conventional, or metaphoric gestures, were not included in these analyses since we did not have a theoretically motivated reason for why these categories would relate to children's spatial performance. Only task-relevant gestures were included. For example, if a child pointed at the timer provided to the dyad, this would not be included in the analysis.

Parental and child spatial gestures were those that were accompanied with a spatial word in the same utterance. For example, if the parent said *corner* and pointed to the corner of the piece, the word *corner* would be coded as a spatial word, and the deictic gesture accompanying this utterance would be coded as a spatial gesture. The spatial gestures were also categorized according to six spatial categories described above: (1) *dimensional* gestures described the size of a person, place, or thing, e.g., holding two flat hands away from each other to describe big; (2) *spatial feature gestures* included those that described properties of non-dimensional aspects of objects, e.g., pointing to the corner of the puzzle for corner, or tracing the border of the puzzle; (3) *positional and directional gestures* that describe the relative position or direction of an object, person in space, e.g., putting two fist hands together while saying together, or pointing to the top of the puzzle board; (4) *shape gestures* used to describe any 2D or 3D enclosed spaces, e.g., making a trace gesture to highlight a circle for circle; (5) *orientation and transformation gestures* that described the relative orientation or transformation of objects in space, e.g., rotate two pinched fingers to describe turn; and (6) *continuous amount gesture* that described the number of continuous quantities, e.g., using a flat hand to describe length or using a whole hand to cover the entire body of an animal on the puzzle. An independent research assistant coded 10% of the data. Kappa was used to establish reliability. Agreement was strong for all categories—gesture presence (0.85), gesture type (0.87), gesture spatial category (0.80), and speech spatial category (0.84). Other than gesture spatial category which yielded substantial agreement, all other categories revealed almost perfect agreement.

## Results

### Parent Demographics and Child Spatial Performance: Relations to Prematurity

#### Demographics

Table 1 presents the demographic characteristics of PTB and TB children and families. PTB and TB groups did not differ on demographic factors, including child sex, chronological age at test, and parent income and parent education. As would be predicted, PTB had significantly lower gestational age in weeks and lower birth weight than TB.

#### Spatial Performance

Table 1 presents the descriptive statistics for PTB and TB children's CMTT and WPPSI Block Design performance. The two groups did not significantly differ from each other on the spatial measures (see Table 1). Within the PTB group, gestational age in weeks did not correlate with CMTT or WPPSI performance (all *p*'s > 0.10). Thus, in the remainder of the paper, we considered prematurity as a binary factor. Table 2 shows that there were no significant correlations between demographic factors (child sex, age at test, birth weight, parent income, and parent education) and children's CMTT or WPPSI performance. As predicted, family income and parent education were significantly correlated. Children's performance on the two tasks were also correlated—children who did better on CMTT also did better on WPPSI Block Design.

**Table 2:** Correlations between spatial performance and demographic characteristics.

	**Maternal education (years)**	**Family income**	**Chronological age (years)**	**Birth weight (pounds)**	**WPPSI block design**	**CMTT**
Maternal education (years)	—					
Family income	0.338^*^	—				
Chronological age (years)	0.029	0.225	—			
Birth weight (pounds)	0.243	0.028	0.198	—		
WPPSI block design	−0.098	0.166	0.201	0.129	—	
CMTT	0.184	−0.147	0.184	0.190	0.362^*^	—

* *p < 0.05*.

### Variability in the Number and Type of Spatial Language and Gesture in Parents and Children: Relations to Prematurity

#### Speech

Table 3 represents the descriptive statistics for total words and spatial words parents and children produced for the two groups (TB and PTB). See Supplementary Materials for descriptive statistics on spatial word types as a function of prematurity status. Before statistical analysis, counts were transformed using log transformation. There were no significant differences between the two groups of parents or between two groups of children as a function of prematurity status on any of the speech measures. Parent and child total number spatial words were significantly correlated with each other, *r* = 0.445, *p* = 0.004. Since the parents varied in the word tokens they used, in the subsequent analyses the number of word tokens was used as a covariate.

**Table 3:** Descriptive statistics for parent and child overall and spatial words and gestures by prematurity group (PTB, TB).

	**PTB**		**TB**			
***n* = 40**	**Mean**	***SD***	**Mean**	***SD***	***t***	***p***
Parent words	442.55	234.11	406.10	233.84	0.1402	0.889
Parent gestures	20.05	13.39	19.15	16.56	0.0582	0.954
Parent spatial words	30.85	20.67	24.25	14.69	0.249	0.805
Parent spatial gestures	8.70	7.03	6.70	5.41	0.2791	0.782
Child words	141.25	103.07	174.40	108.64	−1.3312	0.191
Child gestures	5.00	4.29	7.50	7.45	−1.0652	0.294
Child spatial words	7.00	9.79	6.40	8.04	0.0259	0.979
Child spatial gestures	0.85	1.27	1.45	1.82	−1.2387	0.223

*Descriptive statistics include mean and standard deviation (SD). Inferential statistics include t- and p-values*.

#### Gesture

Table 3 represents the descriptive statistics for the gestures and spatial gestures parents and children produced for the two groups (PTB, TB). See Supplementary Materials for descriptive statistics on spatial gesture types as a function of prematurity status. There were no significant differences between the two groups of mothers or two groups of children as a function of prematurity status on any of the gesture measures. Parent and child total number of spatial gestures were also correlated with each other, *r* = 0.452, *p* = 0.003. Since the parents varied in the total number of gestures used, in the subsequent analyses the number of gestures was used as a covariate.

#### Summary

Taken together, there were no overall differences between the two groups (PTB, TB) in terms of overall or spatial word and gesture use. However, more importantly for our purposes, parents and children varied greatly from each other within their groups. Thus, next we explored the predictors of individual variability.

### Role of Child Spatial Language and Gesture Production and Prematurity in Children's Spatial Performance

Next, we asked whether parent and child spatial words and gestures related to children's performance on spatial tasks. We first examined how children's own spatial speech and gesture use were related to their spatial performance and then examined the contribution of parents use of spatial speech and gesture.

#### CMTT

First, we ran a hierarchical regression analysis, taking CMTT score as outcome variable and child spatial words, child spatial gestures, and prematurity group (PTB, TB) as predictors, controlling for the total number of words and total number of gestures (see Table 4). In the first step of the hierarchical regression, we included prematurity, number of words, and number of spatial words as predictors. Only the number of spatial words emerged as a significant predictor. In the second step of the hierarchical regression, we next added number of gestures and number of spatial gestures as additional predictors. In this model, number of spatial gestures emerged as a significant predictor and the significant effect of spatial words disappeared. Finally, there was a significant negative main effect of number of words. Prior analyses showed that interactions between prematurity and spatial words/gestures neither were significant nor increased the model fit and thus were not included in the models. Similarly, prior analysis showed that parental education and age at test also were not significant predictors and did not improve the model and thus these variables were not included in the main model.

**Table 4:** Hierarchical regressions predicting CMTT and WPPSI Block Design scores using prematurity, child words, spatial words, gestures, and spatial gestures.

	**CMTT estimate (SE)**		**WPPSI block design estimate (SE)**	
	**Model 1**	**Model 2**	**Model 1**	**Model 2**
Prematurity	1.21 (0.71)^~^	0.99 (0.68)	0.53 (1.08)	0.58 (1.11)
Total words	−3.31 (1.66)^~^	−3.62 (1.70)^*^	−0.76 (2.67)	−0.22 (2.93)
Total spatial words	2.46 (1.10)^*^	1.58 (1.11)	1.75 (1.73)	1.91 (1.86)
Total gestures		−2.14 (1.31)		−0.79 (1.97)
Total spatial gestures		4.63 (1.98)^*^		−0.13 (3.22)
R-squared	0.18	0.31	0.04	0.05

* *p < 0.05,*

~ *p < 0.10*.

#### Child WPPSI Block Design

We paralleled another two-step hierarchical regression analysis predicting WPPSI Block Design scores using child spatial words, child spatial gestures, and prematurity group, controlling for total number of words and total number of gestures. None of the predictors reached significance (see Table 4).

#### Spatial What Terms

Pruden et al. (2011) focused on a particular subset of spatial words, which specifically referred to spatial features and properties of objects, in their analysis. These words, also referred to as *what* terms, correspond to three of our categories: Dimensional adjectives, Shape terms, and Spatial feature terms. We reran our analyses focusing on this subset of spatial words to examine if the pattern of results changes. In this model, both number of spatial gestures and number of *what* spatial words emerged as positive significant predictors of children's CMTT score, but not WPPSI Block Design (see Table 5).

**Table 5:** Hierarchical regressions predicting CMTT and WPPSI block design scores using prematurity, child words, subset of spatial what words, gestures, and spatial gestures.

	**CMTT estimate (SE)**		**WPPSI block design estimate (SE)**	
	**Model 1**	**Model 2**	**Model 1**	**Model 2**
Prematurity	1.03 (0.67)	0.83 (0.64)	0.60 (1.00)	0.75 (1.05)
Total words	−0.35 (1.04)	−1.17 (1.10)	0.02 (1.60)	0.38 (1.86)
Total spatial *what* words	3.48 (1.59)^*^	3.74 (1.49)^**^	3.38 (2.44)	3.57 (2.51)
Total gestures		−2.93 (1.25)^*^		−1.17 (1.89)
Total spatial gestures		4.50 (1.93)^*^		0.26 (3.12)
R-squared	0.20	0.35	0.09	0.10

** *p < 0.01,*

* *p < 0.05*.

#### Summary

To sum up, number of children's spatial gestures was a unique contributor to their CMTT performance, over and above spatial words, but not to their WPPSI Block Design performance. Relations did not significantly vary as a function of prematurity. However, spatial words specifically focusing on spatial features and properties of objects, i.e., *what* terms, were predictive of CMTT performance, over and above spatial gestures. Overall gesture use and *what* terms specifically both predicted children's mental transformation performance.

### Relation of Parental Spatial Language and Gesture Production to Children's Spatial Performance

Next, we tested whether parental spatial words and gestures would also relate to children's CMMT and WPPSI Block Design scores. Prior studies examining the role of parental input report a mediation model where the role of the parent input on child spatial skill is mediated by children's own spatial language use (Pruden et al., 2011). A mediation model allows testing both direct effect of parental input on child outcomes and any possible indirect effects of parental input on children's outcomes via children's own spatial language use. Based on this prior literature, we similarly used mediation models to test whether parental use of spatial words and gestures would relate to children's CMTT and WPPSI Block Design scores via children's own use of speech and gesture whether children's own gestures would statistically mediate the relation between parent gesture and child outcomes. Another main goal was to see if the relations would differ as a function of prematurity. For these two goals, we used a moderated mediation approach where we examined whether the relations in the mediation differs as a function of prematurity.

Table 6 presents the moderated mediation model focusing on parent and child words—specifically focusing on the subset of spatial words, i.e., *what* terms, which were predictive for children, since all spatial words did not emerge as a significant predictor in the regression analyses (see Table 5). The moderated mediation model analysis found a significant indirect effect of parental spatial words to child CMTT via child spatial words. Parent spatial words were positively related to child spatial words and child spatial words were positively related to CMTT. There was no significant direct effect of parent spatial words on child CMTT. Prematurity did not moderate the reported relations.

**Table 6:** Moderated mediation models for spatial language: effects that are statistically significant are written in bold.

				**95% CI**				
**Type**	**Effect**	**Estimate**	**SE**	**Lower**	**Upper**	**β**	**z**	**p**
Indirect	**Parent spatial words** ⇒ **child spatial words** ⇒ **CMTT**	**1.197**	**0.582**	**0.057**	**2.337**	**0.223**	**2.058**	0.045^*^
Component	**Parent spatial words** ⇒ **child spatial words**	**0.314**	**0.11**	**0.098**	**0.529**	**0.442**	**2.855**	0.004^**^
	**Child spatial words** ⇒ **CMTT**	**3.814**	**1.284**	**1.297**	**6.331**	**0.504**	**2.97**	0.003^**^
Direct	Parent spatial words ⇒ CMTT	−1.071	0.917	−2.867	0.725	−0.199	−1.169	0.243
Total	Parent spatial words ⇒ child spatial words ⇒ CMTT	0.127	0.948	−1.73	1.985	0.024	0.134	0.893
Interaction	Parent spatial words × prematurity ⇒ child spatial words	0.0022	0.22	−0.429	0.433	0.001	0.01	0.992
	Child spatial words × prematurity ⇒ CMTT	2.5714	2.568	−2.462	7.605	0.225	1.001	0.317
	Parent spatial words × prematurity ⇒ CMTT	−0.0844	1.833	−3.677	3.508	−0.008	−0.046	0.963

*IV, independent variable (parent spatial words); mediator (child spatial words); moderator (prematurity); DV, dependent variable (CMTT). Unstandardized estimate, standard error (SE), 95% confidence interval (CI), standardized estimate (β), z- and p-values are reported. Betas are completely standardized effect sizes. Confidence intervals computed with Standard (Delta method).*

* *p < 0.05,*

** *p < 0.01*.

We next conducted another moderated mediation model analysis using parent spatial gestures as the independent variable and children's spatial gestures as the mediator (see Table 7). There was a significant indirect effect of parental spatial gesture to child CMTT via child spatial gesture. Parent spatial gesture use was positively significantly related to child spatial gesture use and child spatial gesture was positively related to CMTT. There was also a direct statistical effect of parent spatial gesture on children's CMTT score. Prematurity did not moderate the reported relations.

**Table 7:** Mediation models for spatial gesture: effects that are statistically significant are written in bold.

				**95% CI**				
**Type**	**Effect**	**Estimate**	**SE**	**Lower**	**Upper**	**β**	**z**	**p**
**Indirect**	**Parent spatial gesture** **⇒** **child spatial gesture** **⇒** **CMTT**	**1.051**	**0.524**	**0.024**	**2.078**	**0.222**	**2.006**	0.045^*^
**Component**	**Parent spatial gesture** **⇒** **child spatial gesture**	**0.32**	**0.096**	**0.132**	**0.508**	**0.506**	**3.33**	**<** 0.001^*^
	**Child spatial gesture** **⇒** **CMTT**	**3.283**	**1.306**	**0.723**	**5.843**	**0.439**	**2.514**	0.012^*^
**Direct**	**Parent spatial gesture** **⇒** **CMTT**	–**2.164**	**0.844**	–**3.82**	**-0.51**	**-0.46**	**-2.57**	0.01^*^
Total	Parent spatial gesture ⇒ child spatial gesture ⇒ CMTT	−1.104	0.813	−2.698	0.49	−0.232	−1.357	0.175
Interaction	Parent spatial gesture × prematurity ⇒ child spatial gesture	0.0588	0.192	−0.318	0.436	0.046	0.306	0.76
	Child spatial gesture × prematurity ⇒ CMTT	0.6313	2.612	−4.488	5.751	0.059	0.242	0.809
	Parent spatial gesture × prematurity ⇒ CMTT	−1.4963	1.688	−4.804	1.811	−0.158	−0.887	0.375

*IV, independent variable (parent spatial gestures); mediator (child spatial gestures); moderator (prematurity); DV, dependent variable (CMTT). Unstandardized estimate, standard error (SE), 95% confidence interval (CI), standardized estimate (β), z, and p-values are reported. Betas are completely standardized effect sizes. Confidence intervals computed with Standard (Delta method).*

* *p < 0.05*.

A larger model which includes both spatial language and gesture revealed a similar pattern of results. Due to modest sample size, we chose to present two separate models. Because children's own spatial words and gestures did not predict WPPSI Block Design performance, we did not conduct moderated mediation for this outcome.

#### Summary

Overall, parental spatial gestures was related to children's CMTT scores via children's own spatial gestures. Similarly, parent spatial words related to children's CMTT scores via children's own spatial words, suggesting a statistical indirect effect. Relations did not vary as a function of prematurity.

## Discussion

Spatial skills not only are central to many daily activities, such as navigation, but also predict achievement in STEM disciplines, over and above verbal and mathematical skills (e.g., Casey et al., 1997; Shea et al., 2001; Wai et al., 2009; Clements and Sarama, 2011; Uttal et al., 2013; Verdine et al., 2014b). Among different spatial skills, mental rotation skills emerge as particularly important as they continue to strengthen over preschool years and reveal significant individual variability (Newcombe et al., 2013). Individual differences in spatial skills emerge early in life, and some children are at a greater risk for falling behind than others. Children born premature are potentially at risk for falling behind in their cognitive development in general and visuospatial development in particular. However, only a few studies examined preterm children's mental rotation skills at an age later than preschool and none focused on the full spectrum of gestational age. The current study compared PTB and TB children's spatial skills (mental rotation and block design), spatial language, and gesture use of PTB and TB children and their parents during a puzzle task. Finally, we examined the relations between parent and child spatial language and gesture and children's mental rotation and block design skills.

With respect to our first question, we did not find group differences between PTB and TB children in terms of their mental rotation and their block design skills. Evidence on the effect of prematurity on spatial skills, specifically on mental rotation, is scarce. Two prior studies that examined and reported differences between PTB and TB's mental rotation skills focused only on children born very premature (Taylor and Jakobson, 2009, 2013). Here, for the first time, we focused on the full spectrum of gestational age and examined two different spatial skills as well as children's performance during a spatial puzzle task. Including late PTB children is significant since late PTBs constitute the majority of the PT births (75%) and studying late PTB children enables us to examine the effect of prematurity without possible medical confounds that extremely or early born PTBs frequently face. Our results are consistent with a recent study by Kizildere et al. (submitted) who examined mental rotation skills in PTB and TB toddlers and did not find significant differences between PTB and TB toddlers in their mental rotation of objects or between their parents in terms of their spatial language input. We also did not find significant differences between children in terms of their spatial language or gesture use during puzzle play. However, it is important to acknowledge the extent of individual variability in PTB children. Although there were no significant differences between two groups, PTB and TB children greatly varied in their spatial skills. Attempting to understand why some PTB children fare better than others might bring more explanatory power than focusing on broad group differences between PTB and TB (Johnson et al., 2018). Thus, our main question was whether the individual variability in children's spatial skills was related to the spatial language and gestures they produce, which in turn is related to the input they receive from their parents.

We again did not observe significant group differences in the spatial words or gestures PTB and TB parents produced during the puzzle play. Recent work emphasizes the role of broad environmental experiences, such as SES, in PTB children's development (Nepomnyaschy et al., 2012). Less is known about the role of prematurity on proximal, specific aspects of parent–child interactions that might promote children's cognitive development. Existing evidence is mixed (e.g., Bilgin and Wolke, 2015). In terms of language input, while parents of PTB and TB did not seem to differ in terms of their overall amount of language input (Salerni et al., 2007; Adams et al., 2018), or spatial language input (Kizildere et al., submitted), differences in the linguistic complexity of the input has been observed (Kizildere et al., submitted). Overall, given the limited number of studies, future work is needed to compare PTB and TB on quantity and quality of parental language input. More specifically, future research should focus on families that vary along the SES continuum. Here we focused on an overall higher SES sample. Parental SES is a strong predictor of parental input (Schwab and Lew-Williams, 2016) and narrow variability in SES might have masked differences in input due to prematurity. Focusing on a wider SES range might bring greater variability in the input.

Children's use of spatial words (particularly spatial words referring to object dimensions, features, i.e., *what* terms) and use of spatial gestures during spatial puzzle play was related to their performance on the mental rotation task. Importantly, children's spatial language and gesture production did not differ based on prematurity status. In line with the arguments on the close relationship between spatial language and spatial cognition, our results add to the accumulating evidence on this link (e.g., Balcomb et al., 2011; Miller and Simmering, 2018; Simms and Gentner, 2019; Turan et al., 2021). We also provided further evidence that not only spatial language but also spatial gestures produced in another task are associated with children's mental transformation performance. These findings illustrate that both verbal and gestural spatial language could support spatial cognition.

Our main question was to see whether children's and parents' spatial language and gestures were related to children's performance on spatial tasks. Leveraging a statistical mediation model, we showed that children whose parents provide richer spatial speech and gesture input produce richer speech and gesture themselves—this in turn predicted their performance on a mental transformation task. Spatial skills are malleable (e.g., Uttal et al., 2013). Various environmental factors, such as spatial play (e.g., blocks, puzzles) or spatial language (e.g., hearing words about spatial relations), correlate with better spatial skills and also improve spatial skills (e.g., Levine et al., 2012; Verdine et al., 2014a,b; Bower et al., 2020; Casasola et al., 2020). Our findings are in line with the general prior literature as well as with specific studies focusing on spatial language use. Pruden et al. (2011) similarly showed that spatial language focusing on spatial features and properties of objects—also referred to as *what* terms—predicted children's performance on a mental transformation, but not on block design tasks. Our results are consistent with Pruden et al. (2011) who similarly reported relations to mental transformation, but not block design. Differential relations might be because successfully completing the mental transformation task requires verbalizing or highlighting a wider range of spatial features and relations as compared to block design where children need to copy patterns consisting of only a small array of spatial elements. Our results add to the existing results by showing that it is specifically the subset of spatial words, and not all spatial words, that related to children's spatial performance in a mental transformation task.

Our results, for the first time, showed unique contribution of spatial gestures to children's mental transformation performance, over and above spatial language. We showed that spatial gestures of children produced during puzzle play uniquely predicted children's performance on a mental transformation task, over and above spatial speech they produced. Gesturing during a mental transformation task is related to better performance on the same task (Ehrlich et al., 2006). We also showed that the spatial gestures children produced were tightly linked to their parents' spatial gestures. To put differently, the more spatial gestures parents produced, the more spatial gestures children produced which in turn predicted better mental transformation performance. Little is known about parental gesture about specific topics, and less is known about parental spatial gestures in term or in preterm children. Spatial gestures might be better suited to capture continuous features of space than speech and thus might be particularly enriching for children's spatial thinking. Indeed, prior work showed that parental spatial gestures when children were 12–42 months of age predicted children's concurrent use of spatial language over and above parental spatial talk and overall talk (Cartmill et al., 2010). Our results are consistent in that we find concurrent relations between parents and children in their spatial language and gesture use. Here, we add to earlier findings, showing that the role of parental gestures extends beyond the same task to children's performance on independent spatial tasks. Future work should examine whether the specific role of spatial gestures extend beyond STEM-related tasks. For example, given the possible relations between visuospatial skill and gesture production during narrative tasks (Hostetter and Alibali, 2007), one could predict spatial skills to have wider implications.

Our results suggest that different kinds of spatial information expressed in speech and gesture might differentially relate to children's spatial thinking. Speech and gesture might provide different affordances for expressing spatial relations. Speech focusing on spatial features and properties of objects—also referred to as *what* terms—might most effectively highlight spatial properties of objects for speakers and listeners. However, above and beyond static features of objects, due to its dynamic nature in space, gestures expressing all different kinds of spatial relations—including *what* but also *where* terms—might better highlight continuous features of space. Indeed, gesture is tightly linked to spatial thinking. Speakers frequently rely on gestures when they are providing spatial information, ranging from navigating through space to expressing spatial relations in organic chemistry (e.g., Emmorey et al., 2000; Stieff et al., 2016). Speakers are more likely to gesture with spatial words than non-spatial words (Krauss, 1998; Alibali, 2005). Children similarly rely on gestures to convey spatial information and frequently to express information not expressed in speech (Ehrlich et al., 2006; Sauter et al., 2012). This might explain why we found that *all* types of spatial gestures predicted children's mental transformation score, but only a subset of spatial words did so. However, it should be acknowledged that differential relations of speech and gesture to children's performance might also be related to limited number of spatial gestures produced by parents and children. In particular, only including all gestures might have yielded sufficient variability in our analyses. Future studies should create contexts that will elicit higher number of spatial gestures to address this possibility. Particularly, our results should be replicated with other tasks tapping onto a wider range of spatial skills (e.g., dynamic spatial transformation, penetrative thinking), such as tangrams, cross sections, or paper folding, which might vary in the degree to which they rely on spatial language and gestures.

Prematurity did not moderate any of the relations between parent and child spatial language and gesture use and child spatial skill. Some argue that prematurity might present a plasticity factor. PTB children might be more susceptible to environmental exposures than TB children. According to the differential susceptibility hypothesis, PTB may be more susceptible not only to the consequences of negative environmental exposures but also to the benefits of positive ones (Shah et al., 2013; Gueron-Sela et al., 2015). Some posit that neuronal plasticity may partially account for PTB's susceptibility to both negative and positive exposures (DeMaster et al., 2019). However, little is known about whether this theory can be extended to specific parent–child interactions that predict PTB children's cognitive outcomes. Although we failed to find significant differences in the strength of the associations between input and child outcomes, one could argue that our results are not inconsistent with the differential susceptibility hypothesis. PTB children who performed well could have done so because of the rich spatial input of the parents. In other words, it is possible that PTB children initially fell behind but did leverage the input to catch up with their TB peers. Future work examining longitudinal relations between parent input and child outcomes might better answer questions regarding differential susceptibility. Only one study by Kizildere et al. (submitted) that we know of examined the role of parental spatial input in PTB children, focusing on a younger age group. Their results similarly did not show relations between input and performance in a mental rotation task to vary by prematurity. Given our limited sample size, the current study might be underpowered to detect interaction effects. Future work with larger sample sizes is also needed to see whether the role of parent–child interactions on PTB children's development vary along the gestational age continuum. Finally, it is also possible that the profiles of plasticity exhibited by PTB children are system specific (Stevens and Neville, 2009). Future studies should examine whether the role of parents varies across different areas of development, such as language development vs. spatial development. Our data were cross-sectional and thus the study does not warrant causal inference. Experimental manipulations of parent spatial gesture and language are needed to be able to state causal effects of parents on children's spatial performance. Taken together, our findings highlight the importance of considering the role environmental factors, above and beyond biological risk factors. Many interventions for PTs focus on prenatal and early postnatal life (e.g., breastfeeding), and formal follow-ups focus primarily on early PTs (Benzies et al., 2013). Future efforts to best support PTB children would benefit from better understanding the role of the most active ingredient of children's daily experiences—their parents.

In sum, this is one of the first studies that examined mental transformation skills of PTB children using a full spectrum of gestational age. Further, this is also the first study to examine relations between spatial language and gesture by parents and children to children's spatial performance in TB or in PTB children. Our results show that regardless of developmental history (specifically prematurity), both parental spatial language and spatial gesture use relate to children's spatial performance via children's own use of spatial language and gestures. Our results raise the possibility that leveraging the input parents provide may carry important consequences for children's long-term achievement.

## Data Availability Statement

The raw data supporting the conclusions of this article will be made available by the authors, without undue reservation.

## Ethics Statement

The studies involving human participants were reviewed and approved by University of Iowa's Institutional Review Board. Written informed consent to participate in this study was provided by the participants' legal guardian/next of kin.

## Author Contributions

ÖED and TG conceptualized the study and worked on the final version of the manuscript. ÖED wrote the manuscript. PN collected the data. SC-S coded speech and gestures. SC-S and PN prepared the data for analysis. ÖED and SC-S analyzed the data. All authors contributed to the article and approved the submitted version.

## Conflict of Interest

The authors declare that the research was conducted in the absence of any commercial or financial relationships that could be construed as a potential conflict of interest.

## References

[B1] Adams K. A. Marchman V. A. Loi E. C. Ashland M. D. Fernald A. Feldman H. M. (2018). Caregiver talk and medical risk as predictors of language outcomes in full term and preterm toddlers. Child Dev. 89, 1674–1690. 10.1111/cdev.12818 28452393 PMC5660670

[B2] Alibali M. W. (2005). Gesture in spatial cognition: expressing, communicating, and thinking about spatial information. Spat. Cogn. Comput. 5, 307–331. 10.1207/s15427633scc0504_2

[B3] Anderson P. Doyle L. W. Victorian Infant Collaborative Study Group (2003). Neurobehavioral outcomes of school-age children born extremely low birth weight or very preterm in the 1990s. JAMA 289, 3264–3272. 10.1001/jama.289.24.3264 12824207

[B4] Assel M. A. Landry S. H. Swank P. Smith K. E. Steelman L. M. (2003). Precursors to mathematical skills: examining the roles of visual-spatial skills, executive processes, and parenting factors. Appl. Dev. Sci. 7, 27–38. 10.1207/S1532480XADS0701_3

[B5] Balcomb F. Newcombe N. S. Ferrara K. (2011). Finding where and saying where: developmental relationships between place learning and language in the first year. J. Cogn. Dev. 12, 315–331. 10.1080/15248372.2010.544692

[B6] Belsky J. Bakermans-Kranenburg M. J. Van IJzendoorn M. H. (2007). For better and for worse: differential susceptibility to environmental influences. Curr. Dir. Psychol. Sci. 16, 300–304. 10.1111/j.1467-8721.2007.00525.x

[B7] Benbow C. P. Lubinski D. Shea D. L. Eftekhari-Sanjani H. (2000). Sex differences in mathematical reasoning skill at age 13: their status 20 years later. Psychol. Sci. 11, 474–480. 10.1111/1467-9280.00291 11202492

[B8] Benzies K. M. Magill-Evans J. E. Hayden K. A. Ballantyne M. (2013). Key components of early intervention programs for preterm infants and their parents: a systematic review and meta-analysis. BMC Pregnancy Childbirth 13:S10. 10.1186/1471-2393-13-s1-s10 23445560 PMC3561170

[B9] Bilgin A. Wolke D. (2015). Maternal sensitivity in parenting preterm children: a meta-analysis. Pediatrics 136, e177–e193. 10.1542/peds.2014-3570 26034249

[B10] Blencowe H. Cousens S. Oestergaard M. Z. Chou D. Moller A.-B. Narwal R.. (2012). National, regional, and worldwide estimates of preterm birth rates in the year 2010 with time trends since 1990 for selected countries: a systematic analysis and implications. Lancet 379, 2162–2172. 10.1016/S0140-6736(12)60820-4 22682464

[B11] Bower C. Zimmermann L. Verdine B. Toub T. S. Islam S. Foster L.. (2020). Piecing together the role of a spatial assembly intervention in preschoolers' spatial and mathematics learning: influences of gesture, spatial language, and socioeconomic status. Dev. Psychol. 56, 686–698. 10.1037/dev0000899 32134293

[B12] Breslau N. Chilcoat H. Deldotto J. Andreski P. Brown G. (1996). Low birth weight and neurocognitive status at six years of age. Biol. Psychiatry 40, 389–397. 10.1016/0006-3223(95)00399-1 8874840

[B13] Cannon J. Levine S. Huttenlocher J. (2007). A System for Analyzing Children and Caregivers' Language About Space in Structured and Unstructured Contexts. Technical Report, Spatial Intelligence and Learning Center (SILC).

[B14] Cartmill E. Pruden S. M. Levine S. C. Goldin-Meadow S. Center S. I. L. (2010). The role of parent gesture in children's spatial language development, in Proceedings of the 34th Annual Boston University Conference on Language Development (Somerville, MA: Cascadilla Press), 70–77.

[B15] Cartmill E. A. Demir Ö. E. Goldin-Meadow S. (2012). Studying gesture, in Research Methods in Child Language: A Practical Guide, ed E. Hoff (Hoboken, NJ: Blackwell Publishing Ltd.), 208–225. 10.1002/9781444344035.ch14

[B16] Casasola M. (2005). Can language do the driving? The effect of linguistic input on infants'categorization of support spatial relations. Dev. Psychol. 41, 183–192. 10.1037/0012-1649.41.1.183 15656748 PMC2696172

[B17] Casasola M. Wei W. S. Suh D. D. Donskoy P. Ransom A. (2020). Children's exposure to spatial language promotes their spatial thinking. J. Exp. Psychol. 149, 1116–1136. 10.1037/xge0000699 32212765

[B18] Casey M. B. Nuttall R. Pezaris E. (1997). Mediators of gender differences in mathematics college entrance test scores: a comparison of spatial skills with internalized beliefs and anxieties. Dev. Psychol. 33, 669–680. 10.1037/0012-1649.33.4.669 9232382

[B19] Chawanpaiboon S. Vogel J. P. Moller A.-B. Lumbiganon P. Petzold M. Hogan D.. (2019). Global, regional, and national estimates of levels of preterm birth in 2014: a systematic review and modelling analysis. Lancet Glob. Health 7, e37–e46. 10.1016/S2214-109X(18)30451-0 30389451 PMC6293055

[B20] Christodoulou J. Johnson S. P. Moore D. M. Moore D. S. (2016). Seeing double: 5-month-olds' mental rotation of dynamic, 3D block stimuli presented on dual monitors. Infant Behav. Dev. 45, 64–70. 10.1016/j.infbeh.2016.09.005 27744109

[B21] Clements D. H. Sarama J. (2011). Early childhood mathematics intervention. Science 333, 968–970. 10.1126/science.1204537 21852488

[B22] Dall'Oglio A. M. Rossiello B. Coletti M. F. Bultrini M. De Marchis C. Rava L.. (2010). Do healthy preterm children need neuropsychological follow-up? Preschool outcomes compared with term peers. Dev. Med. Child Neurol. 52, 955–961. 10.1111/j.1469-8749.2010.03730.x 20722666

[B23] Davis D. W. Burns B. M. Wilkerson S. A. Steichen J. J. (2005). Visual perceptual skills in children born with very low birth weights. J. Pediatr. Health Care 19, 363–368. 10.1016/j.pedhc.2005.06.005 16286222

[B24] DeMaster D. Bick J. Johnson U. Montroy J. J. Landry S. Duncan A. F. (2019). Nurturing the preterm infant brain: leveraging neuroplasticity to improve neurobehavioral outcomes. Pediatr. Res. 85, 166–175. 10.1038/s41390-018-0203-9 30531968

[B25] Demir Ö. E. Rowe M. L. Heller G. Goldin-Meadow S. Levine S. C. (2015). Vocabulary, syntax, and narrative development in typically developing children and children with early unilateral brain injury: early parental talk about the there-and-then matters. Dev. Psychol. 51, 161–175. 10.1037/a0038476 25621756 PMC4307606

[B26] Demir-Lira Ö. E. Aktan-Erciyes A. Göksun T. (2019). New insights from children with early focal brain injury: lessons to be learned from examining STEM-related skills. Dev. Psychobiol. 61, 477–490. 10.1002/dev.21847 30942517

[B27] Dessalegn B. Landau B. (2008). More than meets the eye: the role of language in binding and maintaining feature conjunctions. Psychol. Sci. 19, 189–195. 10.1111/j.1467-9280.2008.02066.x 18271868

[B28] Ehrlich S. B. Levine S. C. Goldin-Meadow S. (2006). The importance of gesture in children's spatial reasoning. Dev. Psychol. 42, 1259–1268. 10.1037/0012-1649.42.6.1259 17087558

[B29] Emmorey K. Tversky B. Taylor H. A. (2000). Using space to describe space: perspective in speech, sign, and gesture. Spat. Cogn. Comput. 2, 157–180. 10.1023/A:1013118114571

[B30] Esbjørn B. H. Hansen B. M. Greisen G. Mortensen E. L. (2006). Intellectual development in a Danish cohort of prematurely born preschool children: specific or general difficulties? J. Dev. Behav. Pediatr. 27, 477–484. 10.1097/00004703-200612000-00004 17164620

[B31] Estes D. (1998). Young children's awareness of their mental activity: the case of mental rotation. Child Dev. 69, 1345–1360. 10.2307/1132270 9839420

[B32] Foster-Cohen S. H. Friesen M. D. Champion P. R. Woodward L. J. (2010). High prevalence/low severity language delay in preschool children born very preterm. J. Dev. Behav. Pediatr. 31, 658–667. 10.1097/DBP.0b013e3181e5ab7e 20613625

[B33] Frick A. Möhring W. (2013). Mental object rotation and motor development in 8- and 10-month-old infants. J. Exp. Child Psychol. 115, 708–720. 10.1016/j.jecp.2013.04.001 23708734

[B34] Frick A. Möhring W. Newcombe N. S. (2014). Picturing perspectives: development of perspective-taking abilities in 4-to 8-year-olds. Front. Psychol. 5:386. 10.3389/fpsyg.2014.00386 24817860 PMC4012199

[B35] Gentner D. (2016). Language as cognitive tool kit: how language supports relational thought. Am. Psychol. 71, 650–657. 10.1037/amp0000082 27977235

[B36] Gueron-Sela N. Atzaba-Poria N. Meiri G. Marks K. (2015). The caregiving environment and developmental outcomes of preterm infants: diathesis stress or differential susceptibility effects? Child Dev. 86, 1014–1030. 10.1111/cdev.12359 25875941

[B37] Halpern D. F. Benbow C. P. Geary D. C. Gur R. C. Hyde J. S. Gernsbacher M. A. (2007). The science of sex differences in science and mathematics. Psychol. Sci. Public Interest. 8, 1–51. 10.1111/j.1529-1006.2007.00032.x 25530726 PMC4270278

[B38] Hermer-Vazquez L. Moffet A. Munkholm P. (2001). Language, space, and the development of cognitive flexibility in humans: the case of two spatial memory tasks. Cognition 79, 263–299. 10.1016/S0010-0277(00)00120-7 11165214

[B39] Hostetter A. B. Alibali M. W. (2007). Raise your hand if you're spatial: Relations between verbal and spatial skills and gesture production. Gesture 7, 73–95. 10.1075/gest.7.1.05hos

[B40] Johnson S. Waheed G. Manktelow B. N. Field D. J. Marlow N. Draper E. S.. (2018). Differentiating the preterm phenotype: distinct profiles of cognitive and behavioral development following late and moderately preterm birth. J. Pediatr. 193, 85–92. 10.1016/j.jpeds.2017.10.002 29254758

[B41] Kersh J. Casey B. M. Young J. M. (2008). Research on spatial skills and block building in girls and boys, in Contemporary Perspectives on Mathematics in Early Childhood Education, eds O. Saracho and B. Spodek (Charlotte, NC: IAP), 233–251.

[B42] Kisa Y. D. Aktan-Erciyes A. Turan E. Göksun T. (2019). Parental use of spatial language and gestures in early childhood. Br. J. Dev. Psychol. 37, 149–167. 10.1111/bjdp.12263 30069900

[B43] Kizildere E. Kobaş M. Dogan I. Aktan-Erciyes A. Demir-Lira Ö. E. Akman I. (submitted). Parents' spatial language supports preterm full-term toddlers' mental rotation.

[B44] Krauss R. M. (1998). Why do we gesture when we speak? Curr. Dir. Psychol. Sci. 7, 54–54. 10.1111/1467-8721.ep13175642

[B45] Laski E. V. Casey B. M. Yu Q. Dulaney A. Heyman M. Dearing E. (2013). Spatial skills as a predictor of first grade girls' use of higher level arithmetic strategies. Learn. Individ. Differ. 23, 123–130. 10.1016/j.lindif.2012.08.001

[B46] Levine S. C. Goldin-Meadow S. Carlson M. T. Hemani-Lopez N. (2018). Mental transformation skill in young children: the role of concrete and abstract motor training. Cogn. Sci. 42, 1207–1228. 10.1111/cogs.12603 29528134

[B47] Levine S. C. Huttenlocher J. Taylor A. Langrock A. (1999). Early sex differences in spatial skill. Dev. Psychol. 35, 940–949. 10.1037/0012-1649.35.4.940 10442863

[B48] Levine S. C. Ratliff K. R. Huttenlocher J. Cannon J. (2012). Early puzzle play: a predictor of preschoolers' spatial transformation skill. Dev. Psychol. 48, 530–542. 10.1037/a0025913 22040312 PMC3289766

[B49] Loewenstein J. Gentner D. (2005). Relational language and the development of relational mapping. Cogn. Psychol. 50, 315–353. 10.1016/j.cogpsych.2004.09.004 15893523

[B50] Lowe J. R. Erickson S. J. Maclean P. Schrader R. Fuller J. (2012). Association of maternal scaffolding to maternal education and cognition in toddlers born preterm and full term. Acta Paediatr. 102, 72–77. 10.1111/apa.12037 23009657 PMC3566569

[B51] Marlow N. Hennessy E. M. Bracewell M. A. Wolke D. (2007). Motor and executive function at 6 years of age after extremely preterm birth. Pediatrics 120, 793–804. 10.1542/peds.2007-0440 17908767

[B52] McGrath M. Sullivan M. (2002). Birth weight, neonatal morbidities, and school age outcomes in full-term and preterm infants. Issues Compr. Pediatr. Nurs. 25, 231–254. 10.1080/01460860290042611 12542885

[B53] Miller H. E. Andrews C. A. Simmering V. R. (2020). Speech and gesture production provide unique insights into young children's spatial reasoning. Child Dev. 91, 1934–1952. 10.1111/cdev.13396 32720714

[B54] Miller H. E. Patterson R. Simmering V. R. (2016). Language supports young children's use of spatial relations to remember locations. Cognition 150, 170–180. 10.1016/j.cognition.2016.02.006 26896902 PMC4822520

[B55] Miller H. E. Simmering V. R. (2018). Children's attention to task-relevant information accounts for relations between language and spatial cognition. J. Exp. Child Psychol. 172, 107–129. 10.1016/j.jecp.2018.02.006 29604505 PMC5902415

[B56] Moore D. S. Johnson S. P. (2008). Mental rotation in human infants: a sex difference. Psychol. Sci. 19, 1063–1066. 10.1111/j.1467-9280.2008.02200.x 19076473 PMC2651884

[B57] Neel M. L. M. Stark A. R. Maitre N. L. (2018). Parenting style impacts cognitive and behavioural outcomes of former preterm infants: a systematic review. Child Care Health Dev. 44, 507–515. 10.1111/cch.12561 29575031 PMC6005730

[B58] Nepomnyaschy L. Hegyi T. Ostfeld B. M. Reichman N. E. (2012). Developmental outcomes of late-preterm infants at 2 and 4 years. Matern. Child Health J. 16, 1612–1624. 10.1007/s10995-011-0853-2 21769587

[B59] Newcombe N. S. (2020). The puzzle of spatial sex differences: current status and prerequisites to solutions. Child Dev. Perspect. 14, 251–257. 10.1111/cdep.12389

[B60] Newcombe N. S. Uttal D. H. Sauter M. (2013). Spatial development, in Oxford Library of Psychology. The Oxford Handbook of Developmental Psychology, Vol. 1, Body and Mind, ed P. D. Zelazo (Oxford: Oxford University Press), 564–590. 10.1093/oxfordhb/9780199958450.013.0020

[B61] Nomura Y. Halperin J. M. Newcorn J. H. Davey C. Fifer W. P. Savitz D. A.. (2008). The risk for impaired learning-related abilities in childhood and educational attainment among adults born near-term. J. Pediatr. Psychol. 34, 406–418. 10.1093/jpepsy/jsn092 18794190 PMC2722131

[B62] Nord C. Edwards B. Andreassen C. Green J. L. Wallner-Allen K. (2006). Early Childhood Longitudinal Study, Birth Cohort (ECLS-B), User's Manual for the ECLS-B Longitudinal 9-Month−2-Year Data File and Electronic Codebook (NCES 2006–046). National Center for Education Statistics.

[B63] Okamoto-Barth S. Call J. (2008). Tracking and inferring spatial rotation by children and great apes. Dev. Psychol. 44, 1396–1408. 10.1037/a0012594 18793071

[B64] Polinsky N. Perez J. Grehl M. McCrink K. (2017). Encouraging spatial talk: using children's museums to bolster spatial reasoning. Mind Brain Educ. 11, 144–152. 10.1111/mbe.12145 29422944 PMC5798627

[B65] Pruden S. M. Levine S. C. Huttenlocher J. (2011). Children's spatial thinking: does talk about the spatial world matter? Dev. Sci. 14, 1417–1430. 10.1111/j.1467-7687.2011.01088.x 22010900 PMC3372906

[B66] Ralph Y. K. Berinhout K. Maguire M. J. (2020). Gender differences in mothers' spatial language use and children's mental rotation abilities in Preschool and Kindergarten. Dev. Sci. 24:e13037. 10.1111/desc.13037 32931085

[B67] Roseberry S. Göksun T. Hirsh-Pasek K. Golinkoff R. M. (2012). Carving categories in a continuous world: preverbal infants discriminate categorical changes before distance changes in dynamic events. Spat. Cogn. Comput. 12, 231–251. 10.1080/13875868.2011.564338

[B68] Rowe M. L. (2012). A longitudinal investigation of the role of quantity and quality of child-directed speech in vocabulary development. Child Dev. 83, 1762–1774. 10.1111/j.1467-8624.2012.01805.x 22716950 PMC3440540

[B69] Rowe M. L. Goldin-Meadow S. (2009). Differences in early gesture explain SES disparities in child vocabulary size at school entry. Science 323, 951–953. 10.1126/science.1167025 19213922 PMC2692106

[B70] Salerni N. Suttora C. D'Odorico L. (2007). A comparison of characteristics of early communication exchanges in mother-preterm and mother-full-term infant dyads. FirstLanguage 27, 329–346. 10.1177/0142723707081654

[B71] Sauter M. Uttal D. H. Alman A. S. Goldin-Meadow S. Levine S. C. (2012). Learning what children know about space from looking at their hands: the added value of gesture in spatial communication. J. Exp. Child Psychol. 111, 587–606. 10.1016/j.jecp.2011.11.009 22209401 PMC3638086

[B72] Schwab J. F. Lew-Williams C. (2016). Language learning, socioeconomic status, and child-directed speech. Wiley Interdiscip. Rev. Cogn. Sci. 7, 264–275. 10.1002/wcs.1393 27196418 PMC5901657

[B73] Serbin L. A. Zelkowitz P. Doyle A. B. Gold D. Wheaton B. (1990). The socialization of sex-differentiated skills and academic performance: a mediational model. Sex Roles 23, 613–628. 10.1007/BF00289251

[B74] Shah P. E. Robbins N. Coelho R. B. Poehlmann J. (2013). The paradox of prematurity: the behavioral vulnerability of late preterm infants and the cognitive susceptibility of very preterm infants at 36 months post-term. Infant Behav. Dev. 36, 50–62. 10.1016/j.infbeh.2012.11.003 23261789 PMC4235990

[B75] Shea D. L. Lubinski D. Benbow C. P. (2001). Importance of assessing spatial ability in intellectually talented young adolescents. J. Educ. Psychol. 93, 604–614. 10.1037/0022-0663.93.3.604

[B76] Shepard R. N. Metzler J. (1971). Mental rotation of three-dimensional objects. Science 171, 701–703. 10.1126/science.171.3972.701 5540314

[B77] Shusterman A. Lee S. A. Spelke E. S. (2011). Cognitive effects of language on human navigation. Cognition 120, 186–201. 10.1016/j.cognition.2011.04.004 21665199 PMC3572715

[B78] Simms N. K. Gentner D. (2019). Finding the middle: spatial language and spatial reasoning. Cogn. Dev. 50, 177–194. 10.1016/j.cogdev.2019.04.002

[B79] Stevens C. Neville H. (2009). Profiles of development and plasticity in human neurocognition. New Cogn. Neurosci. 165–181.

[B80] Stieff M. Lira M. E. Scopelitis S. A. (2016). Gesture supports spatial thinking in STEM. Cogn. Instr. 34, 80–99. 10.1080/07370008.2016.1145122

[B81] Taylor H. G. Klein N. Minich N. M. Hack M. (2000). Middle-school-age outcomes in children with very low birthweight. Child Dev. 71, 1495–1511. 10.1111/1467-8624.00242 11194251

[B82] Taylor N. Jakobson L. (2009). Mental rotation in preterm children. J. Vis. 9:1061. 10.1167/9.8.1061

[B83] Taylor N. M. Jakobson L. S. (2013). Persisting deficits in mirror-normal discrimination among preterm youth. J. Int. Neuropsychol. Soc. 19, 27–28.

[B84] Turan E. Kobaş M. Göksun T. (2021). Spatial language and mental transformation in preschoolers: does relational reasoning matter? Cogn. Dev. 57:100980. 10.1016/j.cogdev.2020.100980

[B85] Uttal D. H. Miller D. I. Newcombe N. S. (2013). Exploring and enhancing spatial thinking; links to achievement in science, technology, engineering, and mathematics. Curr. Dir. Psychol. Sci. 22, 367–373. 10.1177/0963721413484756

[B86] Verdine B. N. Golinkoff R. M. Hirsh-Pasek K. Newcombe N. S. (2014a). Finding the missing piece: blocks, puzzles, and shapes fuel school readiness. Trends Neurosci. Educ. 3, 7–13. 10.1016/j.tine.2014.02.005

[B87] Verdine B. N. Golinkoff R. M. Hirsh-Pasek K. Newcombe N. S. Filipowicz A. T. Chang A. (2014b). Deconstructing building blocks: preschoolers' spatial assembly performance relates to early mathematical skills. Child Dev. 85, 1062–1076. 10.1111/cdev.12165 24112041 PMC3962809

[B88] Wai J. Lubinski D. Benbow C. P. (2009). Spatial ability for STEM domains: aligning over 50 years of cumulative psychological knowledge solidifies its importance. J. Educ. Psychol. 101, 817–835. 10.1037/a0016127

[B89] Wechsler D. (2012). Wechsler Preschool and Primary Scale of Intelligence, 4th Edn. San Antonio, TX: The Psychological Corporation.

