# Supplementary material for: Spatial Thinking in Term and Preterm-Born Preschoolers: Relations to Parent–Child Speech and Gesture
Source: Front Psychol. 2021 Apr 23;12:651678. doi: 10.3389/fpsyg.2021.651678 (PMC8103033; doi:10.3389/fpsyg.2021.651678)
Supplement: Supplementary file 1 [file Data_Sheet_1.docx]

**Supplementary materials**

*Parent and child spatial word types.*

Parents significantly varied in the type of spatial words they produced. A mixed-model ANOVA with spatial word type as a within and prematurity group as between-subjects factor revealed a main effect of word type, *F*(5, 190) = 114.62, *p* <.001, but not significant effect of prematurity group, *F*(1, 38) = .58, *p* =. 452, or interaction, *F*(5, 190) = 0.797, *p* = .553. Post-hoc analysis showed that parents used Positional and directional terms more frequently than all other categories, all *p*’s < .05. Next two were Spatial feature and Continuous amount terms which were both higher than all of the remaining categories, all *p*’s <.05, the two did not significant differ from each other (*p* = .238). Next were Dimensional adjectives and Orientation and Transformation terms, both of which were more frequent than Spatial terms, both *p’s* <.01, but did not differ from each other, *p* =.304.

For children, a mixed-model ANOVA with spatial word type as a within and prematurity group as between-subjects factor revealed a main effect of word type, *F*(5, 190) = 29.99, *p* <.001, but not significant effect of prematurity group, *F* (1, 38) = 0.01, *p* =. 927, or interaction, *F*(5, 190) = 1.051, *p* = .389. Post-hoc analysis showed that Positional and directional terms and Continuous amount terms were used most frequently (all *p*’s < .001), but the two categories did not significantly differ from each other *p* = .99. The next 4 categories did not differ from each other, all *p*’s > .05, except Dimensional adjectives being more common than Shape terms, *p* = .001.

*Parent and child spatial gesture types.*

Parents significantly varied in the type of spatial gestures they produced. A mixed-model ANOVA with spatial word type as a within and prematurity group as between-subjects factor revealed a main effect of word type, F(5, 190) = 53.44, *p* <.001, but not significant effect of prematurity group, *F* 1, 38) = .10, *p* =. 751, or interaction, *F*(5, 190) = .75, *p* = .589. Post-hoc analysis showed that parents used gestures for Spatial features, Positional and directional gestures and Continuous amount gestures more frequently than all other categories ,all *p*’s < .01. These three categories did not differ from each other, all *p’*s > .05. The remaining three categories also did not vary from each other, all *p*’s > .05.

For children, a mixed ANOVA with spatial word type and prematurity group revealed a main effect of word type, *F*(5, 190) = 10.25, *p* < .001, and a nonsignificant trend for the effect of prematurity group, *F* (1, 38) = 2.92, *p* = .096, and a nonsignificant trending interaction, *F*(5, 190) = 2.09, *p* = .069. Post-hoc analysis showed that Positional and directional terms and Continuous amount terms were used more frequently than the all of the other categories, all *p*’s < .05 – except Dimensional adjectives. The remaining categories did not differ from each other, all *p*’s > .05.
